# Links between household food consumption and nutritional status of children aged 6–59 months: a case study in Burkina Faso

**DOI:** 10.3389/fnut.2025.1696003

**Published:** 2025-11-27

**Authors:** Jean S. R. Kouame, Ella W. R. Compaore, Sonia Blaney, Estelle A. Bambara, Issaka Ouedraogo, Hamidou Sidibe, Mamoudou H. Dicko

**Affiliations:** 1 Laboratory of Biochemistry, Biotechnology, Food Technology and Nutrition (LABIOTAN), Department of Biochemistry-Microbiology, University Joseph KI-ZERBO (UJKZ), Ouagadougou, Burkina Faso; 2 Direction de la Nutrition, Ministère de la Santé, Ouagadougou, Burkina Faso; 3 Secrétariat Technique Chargé de la Multisectorialité Pour la Nutrition (ST-NUT), Ministère de la Santé, Ouagadougou, Burkina Faso; 4 School of Nutrition Faculty of Agricultural and Food Sciences, Université Laval, Québec, QC, Canada; 5 Department of Agricultural and Food Statistics, Ministry of Agriculture, Ouagadougou, Burkina Faso; 6 Davycas International, Ouagadougou, Burkina Faso

**Keywords:** global acute malnutrition, food security, multiple correspondence analysis, Sanguié Province, Burkina Faso

## Abstract

**Introduction:**

Food insecurity is a major challenge in many developing countries and may impede the achievement of Sustainable Development Goal 2 (SDG2). Nearly 15% of Burkina Faso’s population is facing food insecurity. In 2021, 21.6% of children under the age of five (U5) were stunted. This rate remains high. This study aimed to investigate the relationship between Global Acute Malnutrition (GAM) and socio-demographic, economic, and household dietary factors among U5 children in the Sanguié Province in Burkina Faso.

**Methods:**

The method used is based on a quantitative cross-sectional study using primary data on food and nutritional security for 237 children under the age of 5 from 150 households. Data analysis was carried out sequentially: a first univariate descriptive stage was used to characterize the variables studied, with a prevalence of MAG [11.4% (7.35–15.44)], which is higher than the 10% alert threshold set by the WHO; this stage is followed by a bivariate analysis to explore their associations. Finally, a multiple correspondence analysis (MCA) was carried out to identify the independent factors associated with acute malnutrition because of its ability to study complex relationships between variables and to represent their structure in the form of factorial spaces.

**Results and discussion:**

Findings indicate that malnutrition is associated with high household food expenditure; medium dietary diversity; a medium/high demographic dependency ratio; the absence of toilets; and food reserves that cover less than 6 months. These findings highlight the need to strengthen food security by fostering household economic development and to ensure optimal access to improved sanitation.

## 1 Introduction

Food insecurity refers to the lack of regular access, both in quantity and quality, for sufficient, diverse, and nutritious food necessary for proper physical and cognitive development, as well as for maintaining an active and healthy lifestyle (1). Food insecurity is one of the underlying determinants of malnutrition, as it directly impacts individuals’ food intake (2). It currently poses a significant challenge to many developing countries, particularly those committed to achieving Sustainable Development Goal 2.1/2.2 (SDG 2.1/2.2), which aims to end hunger, achieve food security, improve nutrition, and promote sustainable agricultural practices (3).

Available data show that food insecurity issues are critical and threaten public health worldwide. They particularly affect rural populations and the nutritional status of vulnerable groups, especially children under 5 years of age. Malnutrition among children under 5 years has many well-documented consequences: it limits physical and mental health, thereby compromising their well-being and work capacity (4, 5). Yet, child nutrition remains a major public health priority due to its direct link with infant morbidity and mortality; nearly 45–59% of deaths in this age group in low and middle-income countries are attributable to undernutrition (6, 7). At this age and also during the prenatal period, optimal nutrition is crucial for their physical growth and cognitive development, and any deficiency can have irreversible effects. Conversely, good nutrition strengthens the immune system and promotes cognitive development (8–12, 47). Malnutrition, whether acute, chronic, or linked to specific deficiencies, is a complex, multifactorial phenomenon. It results not only from a lack of food but also from the interaction of several structural, socio-economic, health and environmental dimensions (13, 14). With this in mind, the United Nations has proclaimed the period 2016–2025 as the Decade of Nutrition (15).

In sub-Saharan Africa, despite a relative increase in agricultural production, there is still little diversity in household food consumption (16). Regular access to a diversified diet, in sufficient quantity and quality, is a fundamental determinant of child health (17). Smith and Haddad (18) established a positive relationship between household food security and child nutritional status.

Burkina Faso, a landlocked Sahelian country in West Africa, has a population of around 22 million, nearly 80% of whom depend directly on agriculture for their livelihood. This mainly rain-fed sector contributes around a third of the country’s GDP and food security (19). However, agricultural productivity remains low due to dependence upon rainfall, poor mechanization, and soil degradation. Socio-economically, poverty and food insecurity persist, particularly in rural areas, against a backdrop of rapid population growth and increased vulnerability to climate change (20). Strengthening the resilience of agricultural systems, diversifying production, and adding value to local products are essential levers for sustainably improving the country’s food security and socio-economic development (21).

However, in Burkina Faso, child malnutrition remains a major public health problem, with high prevalence of stunting, wasting, and underweight. Agricultural data and nutritional indicators for the last 5 years in Sanguié province, Central West Region (Burkina Faso) were compared with those for the region as a whole and for the three other provinces (Table 1). The analysis revealed a significantly poorer nutritional situation in Sanguié, reflecting increased food insecurity and nutritional vulnerability compared to the other provinces in the region (22–25). While the causes of malnutrition are multifactorial, access and consumption of nutritious food at the household level are considered fundamental determinants (26). However, the precise link between household food consumption patterns and individual child nutritional status remains insufficiently explored in the Burkinabe context (27).

**Table 1:** Comparison of agricultural and nutritional data from Sanguié and other provinces in the Center-West Region of the country.

		Year	2017–2018	2018–2019	2019–2020	2020–2021	2021–2022
Central West Region	Agricultural product	Cereals	354285	547480	462517	555097	474270
		Cash crop	109232	123899	130707	168486	133534
		Other viticultural crops	89929	148634	120145	138187	67164
	Nutritional status	(GAM %)	8.8	9	9	10.2	7.6
Sanguié Province	Agricultural product	Cereals	60407	113158	98664	124458	131629
		Cash crop	11093	14442	18112	28752	20499
		Other viticultural crops	13636	25779	27715	32872	19019
	Nutritional status	(GAM %)	14.2	10	8.1	12.1	10.4
Boulkiemdé Province	Agricultural product	Cereals	55686	113868	83965	83965	112244
		Cash crop	13899	19146	17482	17482	19339
		Other viticultural crops	27028	48547	49956	49956	27053
	Nutritional status	(GAM %)	9	7.6	10.3	11.2	5.6
Sissili Province	Agricultural product	Cereals	157001	217063	180550	180550	132450
		Cash crop	49029	51513	55356	55356	57093
		Other viticultural crops	44427	64807	36242	36242	13757
	Nutritional status	(GAM %)	6.7	7.3	5.9	6.7	5.3
Ziro Province	Agricultural product	Cereals	81190	103391	99338	94143	97946
		Cash crop	35212	38798	39757	37542	36603
		Other viticultural crops	4838	9501	6232	6547	7335
	Nutritional status	(GAM %)	4.5	9.9	11.8	10.3	10.6

This relationship has not yet been carried out, nor has it been shown how it is mediated by socioeconomic factors (e.g., head of household level of education, household size and the household food diversity). Therefore, this study aims to identify statistical associations between household food security indicators and wasting (global acute malnutrition) in children aged 6–59 months in the Sanguié province of Burkina Faso.

As a result, we expect global acute malnutrition in children aged 6–59 months to be significantly associated with factors related to household food security. These results would enable the identification of the main predictive factors of infant malnutrition, prioritizing the relative influence of household food consumption in relation to other determinants, such as maternal education, access to drinking water, or health.

They will be used to design nutritional interventions, enabling resources to be targeted more effectively and decision-makers to improve program planning and policies.

## 2 Methods

### 2.1 Type of study

It is a quantitative and cross-sectional study based on primary data from a food and nutrition security survey.

### 2.2 Study site

Between 2018 and 2021, although the nutritional status (with regard to MAG) of children aged under 5 years living in the province of Sanguié has declined from 14.2 to 12.1% in 2021 (22, 25), still with such prevalence of GAM, it shows a high public health problem according to the World Health Organization (WHO) definition (https://www.who.int/data/nutrition/nlis/info/malnutrition-in-children).

The Sanguié Province, a predominantly rural locality of Burkina Faso, has been selected. Agriculture is the main source of livelihood for households, with high exposure to climate hazards and seasonal food shortages.

### 2.3 Sampling

- The sampling frame used was that of the National Institute of Statistics and Demography (INSD), drawn from the 2019 General Population and Housing Census (RGPH) of Burkina Faso, updated with the village as the primary unit. Villages within the province were selected randomly through systematic sampling with a probability proportional to size (number of households).

The sampling, as described in the first and second stages, is associated with an inclusion probability for each household. The inverse of these inclusion probabilities, in accordance with Horvitz and Thompson (48), constitutes the weighting coefficients used to obtain unbiased estimators. These weighting coefficients were used to extrapolate the results when calculating indicators for all households in the province (number of households, total, etc.).

The household sample size in the Sanguié province, taking into account a non-response rate ***r***, was determined using the following formula (28):


$$n_{i}=\text{Deff}*\frac{t_{\alpha }^{2}p_{i}(1-p_{i})}{(1-r)e^{2}}$$

In this formula, the prevalence “p” was 12.1% [9.6–15.2], the desired precision was 10%, and *α*, the significance level, was *p* = 0.05. The multiplier coefficient Deff refers to the survey effect, which was set at 1.5.

When, for a given province, the number of households obtained does not reach 150, this number is adjusted to be increased to 150 in order to account for specific factors related to nutritional status. This adjustment aims to ensure a sufficient number of observations in each province to estimate the indicators accurately.

In the case of primary units, a fixed number of 10 households per village was selected. It is then possible to calculate the number of villages for the province as follows:


$$m_{i}=\frac{n_{i}\text{ajusted}}{10}$$

On this basis, a first stage of sampling consisted of randomly selecting 15 villages throughout the Sanguié province, then, within each of these villages, 10 households were also randomly selected. Sampling was carried out at the household level. A total of 150 households were selected, and all children aged 6–59 months residing in these households were eligible to participate in the study.

#### 2.3.1 Characteristics of the target population

- Children aged 6–59 months, permanent resident in selected households.
- Location: Urban and rural environment, Sanguié province (Burkina Faso).

#### 2.3.1.1 Selection criteria for children surveyed in the household

##### 2.3.1.1.1 Inclusion criteria

Mandatory conditions for participation.

- Be aged between 6 and 59 months (or the day before the birthday) at the time of recruitment.
- Permanent residence in the selected household.
- Informed consent signed by parent or legal guardian.

#### 2.3.1.1.2 Exclusion criteria

Conditions preventing participation.

- Children with severe congenital malformations that could distort measurements.
- Child suffering from a severe acute illness at the time of the survey (requiring immediate hospitalization).
- Child whose guardian is unable to give informed consent or answer questions.

#### 2.3.1.2 Sampling methodology

- Sampling unit: Household.

##### 2.3.1.2.1 Sample size

A sample of 237 children aged 6–59 months was targeted based on a cluster survey of 150 households.

The clearly defined methodology is a randomized two-stage survey to ensure representativeness.

##### 2.3.1.2.2 Survey period

Data collection took place between February and March 2022.

#### 2.3.2 Observations

##### 2.3.2.1 Anthropometric measurements

- Weight (kg), calibrated electronic scale (precision 100 g) with a SECA battery-operated electronic scale for weighing children under 5, with an accuracy of 100 g. All children were weighed in the nude.
- Height/length (cm), recumbent length for <24 months, standing height for ≥24 months (accuracy 1 mm or 0.1 cm), with a SHORR height board.
- Brachial perimeter (BP): Measured at the left mid-arm with MUAC tape (standard MUAC tape), reading at 1 mm.
- Bilateral foot edema (presence/absence) digital pressure test.
- Derived calculations: Z-score for WHZ, HAZ, WAZ (WHO 2006 standards), and lean/acute indicators.

##### 2.3.2.2 Measurement of household food security variables

- Economic and food variables: Food expenditure class, total food stock duration class, food diversity score, HHS class, food consumption class, food insecurity category (obtained from the household questionnaire).
- Household characteristics: Level of education of head of household, occupation of head of household, number of people in household, number of children under 5 in the household, type of sanitation, dependency ratio (obtained from the household questionnaire).

#### 2.3.3 Data applications

- Estimating prevalence: acute malnutrition, chronic malnutrition, and underweight.
- Establish the link between the nutritional status of children aged 6–59 months and household food security variables: determine predictive indicators to inform decision-makers.

The study adopted a cross-sectional approach based on Standardized Monitoring and Assessment of Relief and Transitions (SMART). This methodology enables a quantitative assessment of the nutritional situation and food security. Data collection was based on a standardized observation protocol, incorporating structured questionnaires validated by technical authorities, as well as precise anthropometric measurements (weight, height, and brachial perimeter), carried out using instruments calibrated according to World Health Organization recommendations (WHO, 2006). This approach aims to guarantee the reliability, comparability, and reproducibility of the data collected on the nutritional status of children aged 6 to 59 months, and on the food security conditions of the households surveyed.

### 2.4 Data collection tools

Data collection was carried out using digital tablets equipped with the CsPro collection application. The following tools were used:

- Digital tablets;
- List of villages;
- List of households to be investigated.

### 2.5 Data collection

Data collection for this survey was carried out in February 2022 using a standardized methodology. The survey protocol was implemented by a joint team from the Ministries of Agriculture and Health, enabling a multi-sectoral approach.

Primary data collection was carried out over a period of 14 days by trained interviewers, using face-to-face interviews assisted by mobile data collection tools. This approach enabled direct digital capture and real-time quality control.

To guarantee data reliability, a rigorous protocol for selecting and training field staff was implemented. Twelve interviewers were recruited, with at least a high school diploma and previous field survey experience. These interviewers were divided into four three-person teams. Preliminary training, lasting five days, covered:

- Expertise in Standardized Monitoring and Assessment of Relief and Transitions (SMART) methodology for nutritional surveys.
- Standardized anthropometric measurement techniques.
- Collection of food security and nutrition indicators.
- Specific use of digital data collection tools developed for the study. All tools and procedures have been pre-tested.

Each surveyor was equipped with a tablet on which a data collection application was installed. This mobile collection application was developed on CsPro.

### 2.6 Data analysis

Following the fieldwork phase, the data were processed and then imported into Stata (statistical software) for tabulation and analysis.

The final database was weighted by calculating weighting coefficients according to the sampling design. All indicators resulting from the analysis are calculated with a 95% confidence threshold.

Data processing was performed using SPSS 25 for descriptive statistics and STATA 18 for modelling.

#### 2.6.1 Statistical and descriptive analysis

Table 2 summarizes and briefly describes the variables used in the study, which are organized into several thematic categories. Each variable is accompanied by its label and modalities.

**Table 2:** Description of the study variables.

Variables	Labelling	Modalities
GAM	Acute malnutrition	0 non-malnourished; 1: Malnourished
Socio-demographic variable		
Gender head	Head of Household’s gender	Female; Male
Age_group_head	Head of household’s age group	Young (0–9 years old); Adult (19–59 years old); Elderly (60 years and over)
Level_education_head	Head of household’s level of education	None; Educated
Dependency _ratio	Demographic ratio	Low (50%); Medium (50–100%); High (100–150%)
Number_children under_5_years	Number of U_5 children	<5 children; > = 5 children
Habitat and hygiene variable		
Type of toilet (availability)	Type of toilet (accessibility)	^1^Yes; No
Economic and food variables		
FEC	Food expenditure class	Less than 50%; 50 à 75%; More than 75%
TFSD	Total food stock duration class (TSDC)	Less than 3 months; 3 to 6 months; More than 6 months
FDS	Food diversity score	Low (1–3); Medium (4–5); High (6–12)
HHS class	HHS class	Moderate hunger (2–3); Little or no (0–1)
FCC	Food consumption class	Poor (0–28); Borderline (28.5–42); Acceptable (42)
HFIAS	Food insecurity category	Food insecurity; Food security

^1^According to WHO criteria (https://www.who.int/data/nutrition/nlis/info/improved-sanitation-facilities-and-drinking-water-sources).

##### 2.6.1.1 Dependent variable

Global Acute Malnutrition (GAM): weight/height < −2 SD of standard values or MUAC between 115 mm and <125 mm or the presence of edema is the dependent variable in this study; it is used to assess the impact of food security factors.

##### 2.6.1.2 Explanatory variables (influential factors)

These variables are grouped as follows.

###### 2.6.1.2.1 Socio-demographic variables

Gender of the head of household: This refers to the gender (male or female) of the person identified as the head of the household, that is, the person who makes the main economic, social, and dietary decisions.

Age of the head of household: The age, in completed years, of the main household manager.

Dependency ratio: It is the ratio between the dependent population (people under 15 and over 64) and the active population (people aged 15 to 64) of the household.

Educational level of head of household: Highest level of formal education attained by the head of household (illiterate, primary, secondary, higher, etc.).

Number of children under 5 in the household: It is the total number of children under 5 living in the household at the time of the survey.

Habitat and hygiene variable (Restroom access only): The proportion of households with functional, safe, and hygienic access to sanitation facilities, whether private, shared, or public (29).

###### 2.6.1.2.2 Food-access related variables

Food expenditure class (FEC): It represents the proportion of total household income spent on food over a given period (usually a week or a month).

Total food stock duration class (TSDC): This indicator measures the length of time a household’s food reserves can cover its needs without new supplies.

Household food diversity score (HFDS): It assesses the number of different food groups consumed by the household over a reference period (often 24 h).

There are generally 12 food groups (cereals; roots and tubers; vegetables; fruit; meat; eggs; fish and seafood; pulses, nuts, and seeds; dairy products; oil and fat; sugar and sweet products; and condiments and beverages). Dietary diversity is a simple and reliable indicator of a household’s degree of food security, in relation to food availability, access, and use (30, 31).

HDDS=Number of food groups consumed by the household over 24 h.

Score HDDS is low (1–3); medium (4–5); and high (6–12).

Household Hunger Scale (HHS) is a standardized indicator widely used to measure the severity of hunger and food insecurity at the household level.

It was developed by the FANTA Project (Food and Nutrition Technical Assistance Project) in collaboration with the World Food Programme (WFP) and USAID.

Household food consumption score (HFCS) was developed by the WFP, and it combines dietary diversity, frequency of consumption, and the nutritional importance of different food groups. Each food group is weighted according to its nutritional value (e.g., protein, energy, and micronutrients).

Food security: Household food security was investigated using the Household Food Insecurity Access Scale (HFIAS), a standardized methodology developed by USAID/FANTA to measure households’ access to food, i.e., their level of food insecurity related to access, using a questionnaire with nine basic questions.

#### 2.6.2 Univariate descriptive analysis

Univariate descriptive analysis examines the distribution of the variables in the study, in particular their distribution, frequency, and main characteristics. In this research, it is used to summarize the data, highlight central trends and dispersion, and is an essential preliminary step before any bivariate or multivariate analysis.

#### 2.6.3 Bivariate analysis

Bivariate analyses were used to assess associations between the dependent and independent variables to identify factors potentially linked to acute malnutrition.

The chi-square (χ^2^) test was used to assess the presence of a statistically significant relationship between each independent variable and acute malnutrition.

The significance of the associations will be assessed at the threshold of 5%.

#### 2.6.4 Multivariate analysis

To identify the factors associated with acute malnutrition, we use a multivariate analysis method that allows for the simultaneous examination of the effects of multiple variables on this condition. To this end, Multiple Correspondence Analysis (MCA) is a particularly suitable statistical tool.

This method highlights the interrelationships between different categorical variables, thus facilitating the detection of groupings based on their statistical proximity (32). It also makes it possible to visualize the relative position of the modalities with respect to each other in a factorial space, thus revealing the underlying structures of the data. This approach enables the extraction of a smaller number of composite dimensions while preserving most of the information contained in the original variables.

## 3 Results

### 3.1 Characteristic of the population

Tables 3, 4 present the socio-economic, demographic, and nutritional data of the sample. Table 3 shows a relatively high prevalence of GAM (11.4%), which is above the WHO alert threshold of 10% and indicates a high public health problem. In Table 4, households are mainly headed by men, accounting for 94.9%. Looking at the age range of the household head, adults account for approximately 70%, indicating their predominance. The results also indicate that the majority of household heads have not received any formal education. The dependency ratio is predominantly low to moderate.

**Table 3:** GAM representation (*n* = 237).

Variable	Modality	Number	Prevalence (%)
GAM	Malnourished children	27	11.4
	Non-malnourished children	210	88.6
	NA	31	
	Total	268	

**Table 4:** Distribution of the food security variables of the study (*n* = 150).

Variable -modalities	Percentage %
Head of household’s gender	
Female	5.06
Male	94.94
Head of household’s age group	
Young	9.7
Adult	70.04
Elderly person	20.25
Head of household’s level of education	
None	66.67
Educated	33.33
Dependency ratio	
Low	42.62
Average	48.52
High	8.86
Number of U_5 children	
< 5 children	74.68
> = 5 children	25.32
Type of toilet	
No	57.81
Yes	42.19
Food expenditure class	
Less than 50% of total expenditures	38.4
50 to 75%	45.57
More than 75%	16.03
Total food stock duration class (TSDC)	
Less than 3 months	11.81
3 to 6 months	23.63
More than 6 months	64.56
Food diversity score	
Low	7.59
Average	71.31
High	21.1
HHS class	
Moderate hunger	8.02
Little or no	91.98
Food consumption class	
Acceptable	59.07
Borderline	34.18
Poor	6.75
Food insecurity category	
Food insecurity	60.34
Food security	39.66

Data show that over 45% of households spend more than half their budget on food. Furthermore, more than 60% of households have sufficient food stocks to cover their needs beyond a period of 6 months, indicating a certain degree of self-sufficiency and food security in the medium term. This could explain household dietary diversity (71.31%), which is generally moderate, and more than half of households (59%) have an acceptable level of food consumption. However, over 40% experience borderline or poor consumption levels.

More than 60% of households are food insecure.

### 3.2 Nutritional status of children under 5

Table 5 highlights the prevalence of malnutrition according to the nutritional indicator and the child’s age. The results show that wasting (an indicator of acute malnutrition) impacts approximately 11.4% of children aged 6 to 59 months, with a 95% confidence interval of [7.35–15.44]. Stunting (low height-for-age), an indicator of chronic malnutrition, increases sharply after 6 months of age, rising from 6.9% among children aged below 6 months to 26.6% among those aged 6–59 months.

**Table 5:** Prevalence of malnutrition by nutritional indicators and groups âge (*n* = 237).

Nutritional indicators	Age group (months)	Prevalence (%)	CI 95%	*n*
Wasting (weight-for-height) GAM	<6	0.0	—	0
	6–59	11.4	[7.35–15.44]	237
	Total	11.4	[7.35–15.44]	237
Stunting (height-for-age)	<6	6.9	[1.91–21.96]	29
	6–59	26.6	[20.96–32.21]	237
	Total	24.4	[19.27–29.60]	266
Underweight (weight-for-age)	<6	6.9	[1.91–21.96]	29
	6–59	21.0	[15.83–26.18]	238
	Total	19.5	[14.73–24.23]	267

Underweight, which reflects both acute and chronic malnutrition, is also more prevalent among older children, affecting 21.01% of them. In summary, Table 5 clearly indicates an increase in malnutrition with child aging.

### 3.3 Bivariate analysis

Table 6 presents a bivariate analysis of acute malnutrition in relation to various socio-demographic and nutritional factors.

**Table 6:** Distribution (%) of global acute malnutrition according to food security factors.

Variable	Modality	Acute malnutrition	
		Malnourished (%)	*P* value
Head of household’s gender	Female	8.3	1.0
	Male	11.5	
Head of household’s age group	Young	13.0	0.9
	Adult	11.4	
	Elderly person	10.4	
Head of household’s level of education	None	11.3	1.0
	Educated	11.3	
Dependency ratio	Low	5.9	0.0
	Average	13.9	
	High	23.8	
Number of U-5 children	< 5 children	10.1	0.4
	> = 5 children	15.0	
Type of toilet	No	10.9	0.9
	Yes	12.0	
Food expenditure class	Less than 50%	12.0	0.2
	50 to 75%	8.3	
	More than 75%	18.4	
Total food stock duration class (TSDC)	Less than 3 months	7.1	0.4
	3 to 6 months	16.0	
	More than 6 months	10.4	
Food diversity score	Low	0.0	0.08
	Average	14.2	
	High	6.0	
HHS class	Moderate hunger	15.7	0.8
	Little or no	11.0	
Food consumption class	Acceptable	10.7	0.9
	Borderline	12.3	
	Poor	12.5	
Food insecurity category	Food insecurity	9.5	0.6
	Food security	12.5	

Indeed, the results of the bivariate analysis reveal two significant variables: the dependency ratio and the dietary diversity. A higher prevalence of acute malnutrition (23.8%) is noted among children living in households with a high dependency ratio (*p* = 0.0). This proportion is lower for children living in households with lower (5.9%) and average (13.9%) demographic ratios.

Households with an average dietary diversity show a 14.2% prevalence of children affected by GAM, whereas those with low dietary diversity report no cases of malnutrition (0%) (*p* = 0.08).

### 3.4 Multivariate analysis

**Figure 1:**
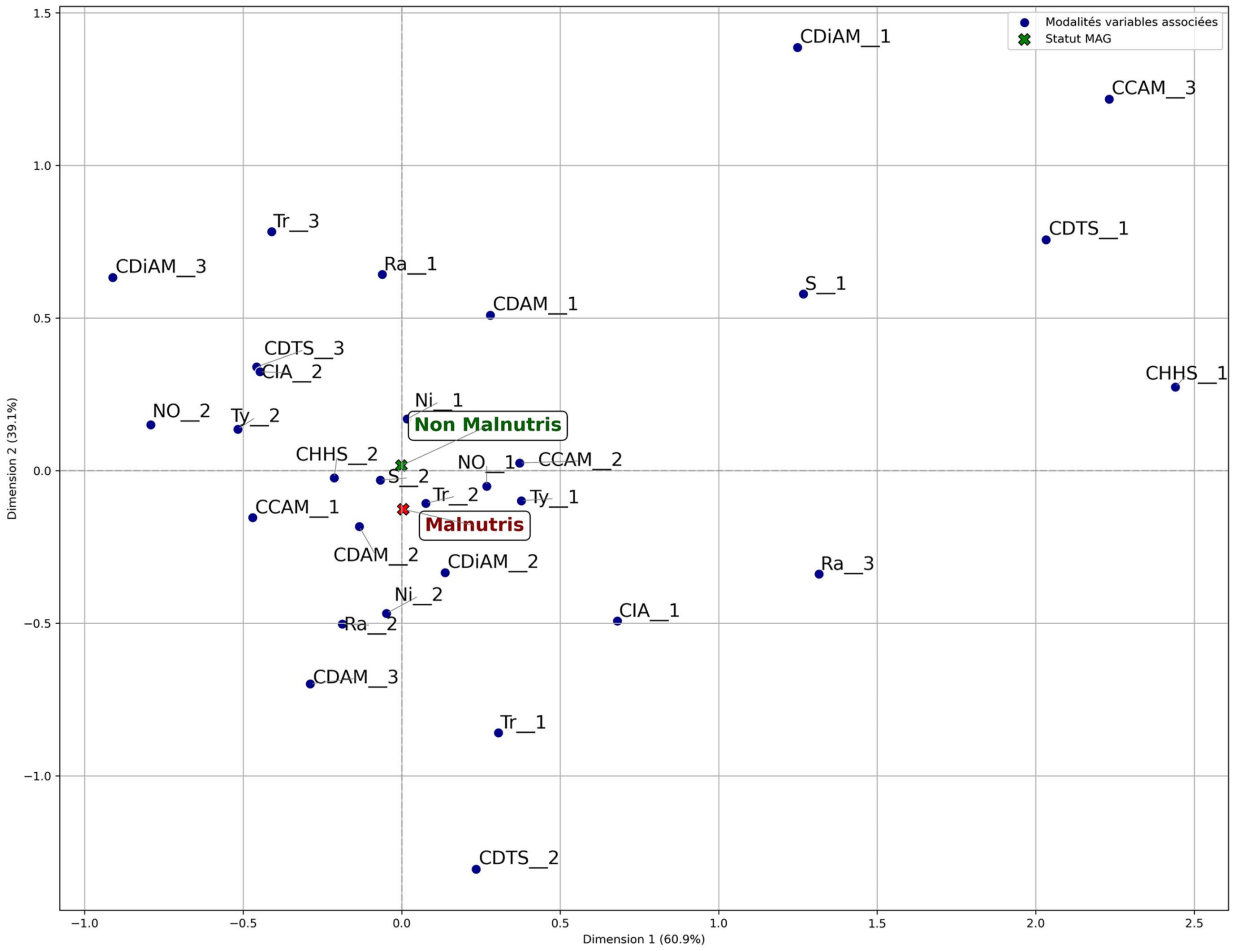
Projection of food security and nutritional status variables for children aged 6–59 months (MCA).

The statistical method Multiple Correspondence Analysis (MCA) was used to analyze the link between certain household food security variables and the GAM. It is based on a graphical representation (Figure 1; Table 7) in which the variable modalities are projected into a reduced-dimensional space.

**Table 7:** Codification of indicators in Figure 1.

Labelling	Code/labelling	Code/modality	Modality
Gender head of household	S	S1	Female
		S2	Male
Age group of head of household	Tr	Tr1	Young
		Tr2	Adult
		Tr3	Elderly person
Head of household’s level of education	Ni	Ni1	None
		Ni2	Educated
Dependency ratio	Ra	Ra1	Low
		Ra2	Average
		Ra3	High
Number of U-5 children	No	No1	<5 enfants
		No2	> = 5 children
Type of toilet	Ty	Ty1	No
		Ty2	Yes
Household food expenditure class	CDAM	CDAM1	Less than 50%
		DDAM2	50 to 75%
		CDAM3	More than 75%
Total food stock duration class (TSDC)	CDTS	CDTS1	Less than 3 months
		CDTS2	3 to 6 months
		CDTS3	More than 6 months
Household Dietary Diversity Score (HDDS)	CDiAM	CDiAM1	Low
		CDiAM2	Average
		CDiAM3	High
HHS class	CHHS	CHHS1	Moderate hunger
		CHHS2	Little or no
Household food consumption class	CCAM	CCAM1	Acceptable
		CCAM2	Borderline
		CCAM3	Poor
Food insecurity category	CIA	CIA1	Food insecurity
		CIA2	Food security

#### 3.4.1 Modalities significantly associated with acute malnutrition

The MCA of this article is made according to the analysis methodology proposed by Moschidis et al. (33). The analysis of the factorial plan shows that the first axis (Horizontal) explains 61.3% of the variations observed. It accounts for most of the inertia (variance). It could represent a socio-economic or nutritional gradient, contrasting households with malnourished children with those with non-malnourished children. Analysis of the projected plan reveals that malnutrition is present in households with the following characteristics.

##### 3.4.1.1 Dependency ratio (Ra)

- Modalities involved: medium and high dependency ratios:

A statistically significant link was found between a higher dependency ratio and a greater prevalence of acute malnutrition. Acute malnutrition affected 13.91% of households with an average dependency ratio (*p* = 0.03) and jumped to 23.81% in those with a high ratio (*p* = 0.03).

##### 3.4.1.2 Household dietary diversity class (CDiAM)

- Modality involved: average household diversity

The data analysis indicates a trend suggesting that moderate dietary diversity is associated with an increased prevalence of acute malnutrition among children. Indeed, 14.2% of children from households with average dietary diversity are malnourished, while the remaining 85.8% are not.

##### 3.4.1.3 Household food expenditure class (CDAM)

- Modality involved: More than 75% of household food expenditures among households allocating over 75% of their total expenditure to food, 18.42% of children are malnourished, while 81.58% are not.

##### 3.4.1.4 Total food stock duration class (TSDC)

- Modality involved: stock duration between 3 and 6 months

The data show that, in households with food stocks covering 3 to 6 months, 16.07% of children are acutely malnourished, compared to 83.93% who are not malnourished.

##### 3.4.1.5 Type of toilet

- Modality involved: absence of toilet (Ty1)

The survey results show that 10.95% of children in households lacking toilets are acutely malnourished, compared to 89.05% who are not.

##### 3.4.1.6 Food insecurity (CIA)

- Modality involved: food insecurity

In households experiencing food insecurity, as measured by the HFIAS, 9.57% of children are malnourished, while 90.43% are not.

#### 3.4.2 Less significant, but notable variable

- Level of education of the head of household:

While households with an educated head show a higher incidence of malnutrition (11.39%), MCA suggests that this is an indirect link, mediated by other factors.

## 4 Discussion

This study aims to identify potential determinants associated with acute malnutrition in children aged 6–59 months in the Sanguié province. There is no direct correlation between low household dietary diversity and children’s nutritional status. This result may seem counterintuitive, as we would generally expect higher rates of malnutrition in young children living in households with limited dietary diversity. However, this could be explained by the fact that in households with low dietary diversity, young children often receive special foods, so household dietary diversity does not reflect what is potentially accessible and given to children under 5. It could be that the number of households classified as “low diversity” is too low, reducing the likelihood of observing cases of malnutrition in this specific group. Finally, unobserved factors such as hygiene practices toward young children could influence these results. The results of this study support previous articles, in particular that of Yessoufou et al. (34) and Hasanah et al. (35). These unobserved factors (hygiene practices and health) play a decisive role in the nutritional status and food security of children under the age of five. Inadequate hygiene, particularly the failure to wash hands with soap, the use of contaminated water, or the unhygienic disposal of excreta, encourages the transmission of pathogens responsible for diarrhoeal and parasitic diseases. These conditions lead to malabsorption of nutrients, loss of appetite, and increased energy requirements, compromising the child’s growth and development.

Improving hygiene practices within households, in particular hand-washing with soap, access to safe drinking water, and adequate sanitation, is, therefore, an essential means of breaking the cycle of infection and malnutrition (36). This convergence of results reinforces the hypothesis that low household dietary diversity, reflecting qualitative food insecurity, is a major determinant associated with infant malnutrition (6–59 months), over and above quantitative aspects of food availability alone. With food expenses accounting for over half of total household expenditures, these households face a precarious food security situation that could negatively affect the nutritional status of children under 5. A study conducted by Jean et al. (37) found that roughly 73% of households in the TONPKI region of Côte d’Ivoire allocate 15 to 45% of their income to food expenses, particularly during the lean season when malnutrition rates are high. Furthermore, Zhang et al. (38) research highlights that the sheer amount spent on food by a household is not synonymous with the consumption of quality food. This result, highlighted by the MCA, underscores the importance of balancing food expenditure and overall living conditions in malnutrition prevention strategies.

In the context of this study, the MCA’s finding of a high demographic dependency ratio linked to acute malnutrition in young children suggests that the substantial number of economically dependent individuals compared to the smaller active population within households, could be a contributing factor. However, the high number of children under and over five places pressure on the household, potentially worsening its food security situation (39). This result could be explained by the increased pressure on household resources, including food, within families carrying a high demographic load. The necessity to allocate food and health resources among numerous household members can result in reduced quantity and quality of nutritional intake available to each child. This structural constraint is especially detrimental to the youngest children, who are more nutritionally vulnerable (40). This is confirmed by the demographic burden faced by some sub-Saharan countries, including Burkina Faso, where a disproportionately large young population compared to working adults and high annual birth rates coincide with limited public spending aimed at improving nutrition (41).

The absence of toilets, as observed in this study, is widely recognized as a significant aggravating factor in malnutrition; finally, more than half of households do not have access to toilets, increasing the risk of diarrheal diseases and, consequently, malnutrition among children.

Ademas et al. (42) noted that the high rate of illness and malnutrition among young children (6–59 month) is due to the absence of toilets and inadequate sanitation services. In sub-Saharan Africa, approximately 88–89% of the population lacks access to toilets, contributing to stunting in 28–49% of children under 5 years of age (43). In the municipality of Karimama (northern Benin), a nutritional survey found that 11.9% of children under five suffered from acute malnutrition, while 39% were affected by chronic malnutrition These results were linked to the lack of a healthy environment, characterized by unsafe water sources, poor household waste management, inadequate handwashing practices, and open defecation (44).

A relationship has been observed between households headed by individuals with limited education and the presence of acute malnutrition in children under five. This explains its less significant link when it is correlated with acute malnutrition in the Sanguié Province (45).

Households with food stocks lasting less than six months show higher rates of acute malnutrition. Food stocks are primarily used to meet daily household needs, provide cash flow through sales when necessary, and enhance the availability of agricultural products that boost dietary diversity (46). This highlights the necessity for households to maintain food stocks exceeding 6 months to successfully cope with the lean season, which may reflect a better ability to adapt to seasonal fluctuations in food availability.

Despite the existence of determinants already well documented in the literature, such as dietary diversity and access to sanitation, this study provides recent, contextualized empirical data for the Sanguié province, an area hitherto little explored in terms of nutrition. This regional contribution provides a relevant baseline for guiding local interventions and situating the nutritional situation in Sanguié in relation to other provinces in Burkina Faso. In addition, the combination of multivariate analysis and MCA gives the study a solid methodological foundation, enabling us to highlight the complex interactions between socio-economic factors, food, and nutritional status. This integrated approach represents significant added value for the design and implementation of nutrition policies and programs adapted to the local context.

## 5 Conclusion

This study examines the links between global acute malnutrition and household socioeconomic and dietary characteristics in Sanguié province, Burkina Faso. MCA has also been used as a method because of its ability to study complex relationships between variables and to represent their structure in the form of factorial spaces. Acute malnutrition is particularly prevalent in households facing a high dependency ratio, with an average dietary diversity, and low food stock (available stocks for less than six months). This is further compounded by a significant food budget weight (over 75% of total household expenses), the lack of basic hygiene infrastructure (toilet), and a high proportion of children under 5 years old within the household. These results confirm that global acute malnutrition is a multifactorial phenomenon, stemming from intricate interactions among food insecurity, precarious socioeconomic conditions, and inadequate health practices.

The fact that most households are headed by uneducated men, coupled with limited financial capacity, may partly account for the persistence of malnutrition in some households.

These results highlight the need for targeted intervention strategies. Interventions integrating improved food availability, nutrition education, and the strengthening of household purchasing power appear to be effective levers for reducing child malnutrition. Approaches combining home fortification with micronutrient powders, the promotion of fortified local foods, dietary diversification, the cultivation of biofortified varieties, and the development of community nutrition gardens are proving particularly promising. In addition, community involvement in the early detection and management of malnutrition, supported by digital technology, as well as the implementation of warning systems, hygiene, and sanitation actions strengthen the nutritional resilience of households. Finally, participatory economic approaches, such as microcredit and community mobilization, help target vulnerable populations. The adoption of these multi-sectoral strategies should be prioritized in order to optimize nutritional gains during the first 1,000 days of life.

## Data Availability

The raw data supporting the conclusions of this article will be made available by the authors, without undue reservation.
